# Crystal structure and Hirshfeld surface analysis of (2*E*)-3-(3-chloro­phen­yl)-1-(3,4-di­meth­oxy­phen­yl)prop-2-en-1-one

**DOI:** 10.1107/S205698901800837X

**Published:** 2018-06-08

**Authors:** S. N. Sheshadri, Zeliha Atioğlu, Mehmet Akkurt, C. S. Chidan Kumar, Ching Kheng Quah, B. P. Siddaraju, M. K. Veeraiah

**Affiliations:** a Department of Chemistry, GSSS Institute of Engineering & Technology for Women, Mysuru 570 016, Karnataka, India; b İlke Education and Health Foundation, Cappadocia University, Cappadocia Vocational College, The Medical Imaging Techniques Program, 50420 Mustafapaşa, Ürgüp, Nevşehir, Turkey; c Department of Physics, Faculty of Sciences, Erciyes University, 38039 Kayseri, Turkey; d Department of Engineering Chemistry, Vidya Vikas Institute of Engineering & Technology, Visvesvaraya Technological University, Alanahalli, Mysuru 570 028, Karnataka, India; e X-ray Crystallography Unit, School of Physics, Universiti Sains Malaysia, 11800 USM, Penang, Malaysia; f Department of Chemistry, Cauvery Institute of Technology, Mandya 571 402, Karnataka, India; g Department of Chemistry, Sri Siddhartha Institute of Technology, Tumkur 572 105, Karnataka, India

**Keywords:** crystal structure, 3,4-di­meth­oxy­phenyl ring, 3-chloro­phenyl ring, Hirshfeld surface analysis, hydrogen contacts, ring motif

## Abstract

In title compound, C_17_H_15_ClO_3_, the dihedral angle between the benzene and chloro­phenyl rings is 18.46 (7)°. In the crystal, mol­ecules are linked by C—H⋯O hydrogen contacts, enclosing an *R* ^2^_2_(14) ring motif, and by a further C—H⋯O hydrogen contact, forming a two-dimensional supra­molecular structure extending along the direction parallel to the *ac* plane. Hirshfeld surface analysis shows that van der Waals inter­actions constitute the major contribution to the inter­molecular inter­actions, with H⋯H contacts accounting for 36.2% of the surface.

## Abstract 2

In the crystal, mol­ecules are linked by C—H⋯O hydrogen contacts, enclosing an 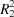
(14) ring motif, and by a further C—H⋯O hydrogen contact, forming a two-dimensional supra­molecular structure extending along the direction parallel to the *ac* plane.

## Chemical context   

Materials exhibiting two photon absorption (TPA) have wide applications such as frequency-up lasing, multi-photon microscopy, three-dimensional fluorescence imaging, eye and sensor protection. Materials with potential non-linear optical (NLO) properties have significant applications in the field of photonics. Chalcone and its derivatives have attracted significant attention in the past few years because of their availability of high optical non-linearities resulting from the significant delocalization of π-conjugated electron clouds throughout the chalcone system, providing a large charge-transfer axis with appropriate substituents on the terminal aromatic rings. The second harmonic generation (SHG) efficiency of these compounds is due to the strong inter­molecular electron–donor–acceptor inter­actions, which may also enhance the non-linear optical (NLO) properties. With the possibility of developing low-cost, large-area and flexible electronic devices, π-conjugated systems have been studied extensively for their optoelectronic properties (Chandra Shekhara Shetty *et al.*, 2016 ▸, 2017 ▸).
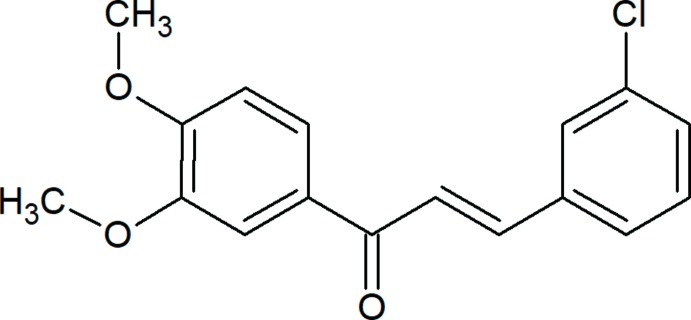


## Structural commentary   

**Figure 1:**
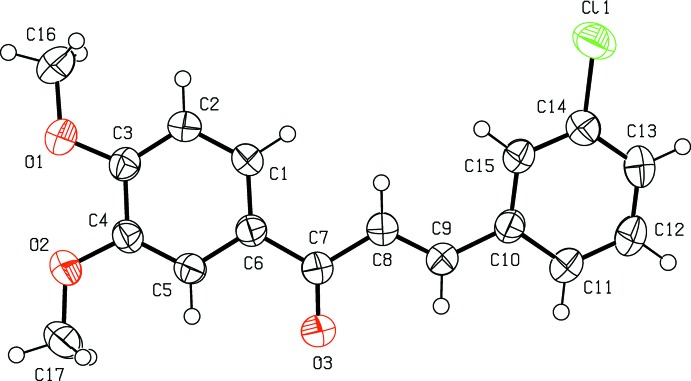
The mol­ecular structure of the title compound, showing the atom labelling and displacement ellipsoids drawn at the 50% probability level.

The mol­ecular structure of the title compound is shown in Fig. 1 ▸. The title compound is constructed from two aromatic rings (chlorophenyl and terminal meth­oxy­phenyl rings), which are linked by a C=C—C(=O)—C enone bridge. Compared to the nearly coplanar arrangement of rings in the title compound, the molecule is twisted substanti­ally [C5—C6—C7—O3 = 3.5 (2) ° and O3—C7—C8—C9 = 10.5 (2) °] about the enone bridge, which may arise from steric repulsion with the *ortho*-O2 atom. Hence, the dihedral angle between the 3,4-meth­oxy­phenyl and chloro­phenyl rings increases to 18.46 (7)°. The C atoms of the meth­oxy groups are close to the plane of their attached ring: deviations of C16 and C17 are 0.252 (2) and 0.038 (2) Å, respectively. The bond lengths and angles are comparable with those in the similar compounds (*E*)-3-(3,4-di­meth­oxy­phen­yl)-1-(1-hy­droxy­naph­th­alen-2­yl)prop-2-en-1-one (Ezhilarasi *et al.*, 2015 ▸), (*E*)-1-(3-bromo­phen­yl)-3-(3,4-di­meth­oxy­phen­yl)prop-2-en-1-one (Esco­bar *et al.*, 2012 ▸) and (*E*)-3-(2-bromo­phen­yl)-1-(3,4-di­meth­oxy­phen­yl)prop-2-en-1-one (Li *et al.*, 2012 ▸).

## Supra­molecular features and Hirshfeld surface analysis   

**Figure 2:**
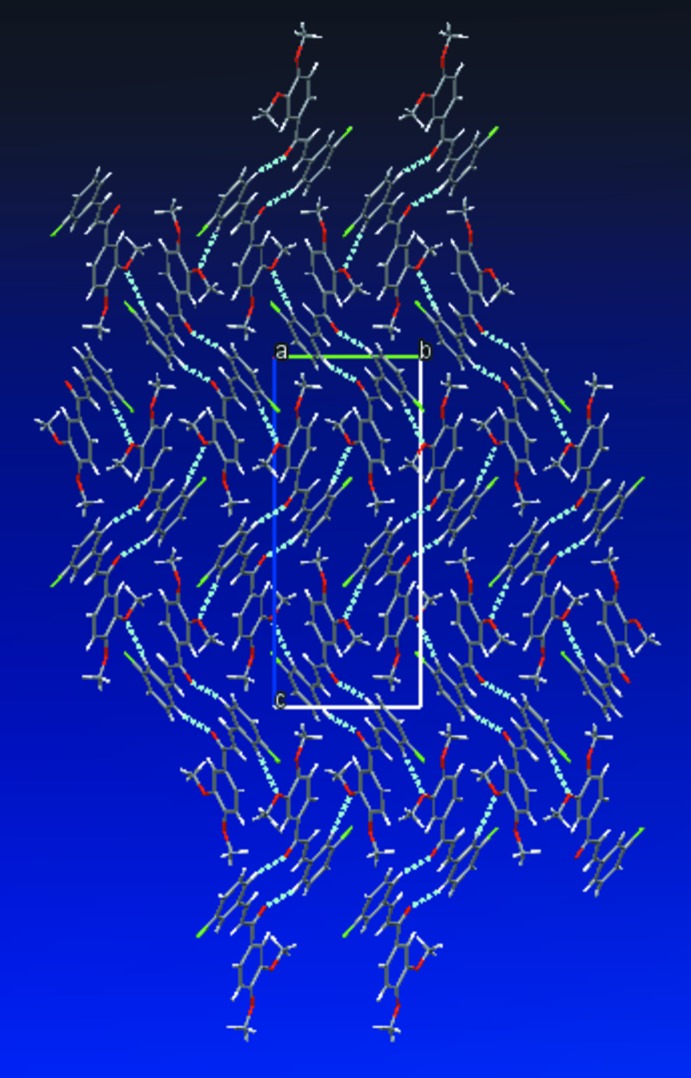
A view along the *a* axis of the crystal packing of the title compound. Inter­molecular inter­actions are shown as dashed lines.

In the crystal, mol­ecules are linked by C—H⋯O hydrogen contacts (Table 1 ▸, Fig. 2 ▸), enclosing an 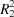
(14) ring motif, and by a further C—H⋯O hydrogen contact, forming a three-dimensional structure extending in the *a*- and *c*-axis directions.

**Figure 3:**
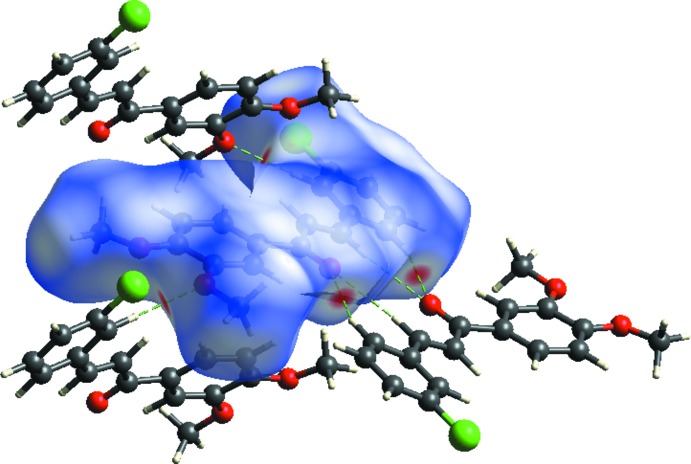
View of the three-dimensional Hirshfeld surface of the title compound mapped over *d*_norm_.

Hirshfeld surfaces and fingerprint plots were generated for the title compound based on the crystallographic information file (CIF) using *CrystalExplorer* (McKinnon *et al.*, 2007 ▸). Hirshfeld surfaces enable the visualization of inter­molecular inter­actions by different colors and color intensity, representing short or long contacts and indicating the relative strength of the inter­actions. Figs. 3 ▸ and 4 ▸ show the Hirshfeld surfaces mapped over *d*_norm_(−0.16 to 1.25 a.u.) and shape-index (−1.0 to 1.0 a.u.).

In Fig. 3 ▸, the spots near atoms O2 and O3 result from the C15—H15*A*⋯O2^ii^ and C11—H11*A*⋯O3^i^ inter­actions significant in the mol­ecule packing of the title compound (Table 1 ▸). Some of the short inter­molecular contacts for the title compound are listed in Table 2 ▸. The Hirshfeld surfaces illustrated in Fig. 3 ▸ also reflect the involvement of different atoms in the inter­molecular inter­actions through the appearance of blue and red regions around the participating atoms, which correspond to positive and negative electrostatic potential, respectively.

**Figure 4:**
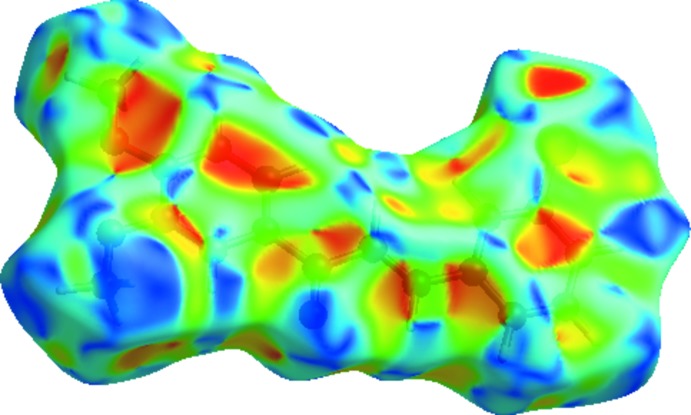
Hirshfeld surface of the title complex plotted over shape-index.

**Figure 5:**
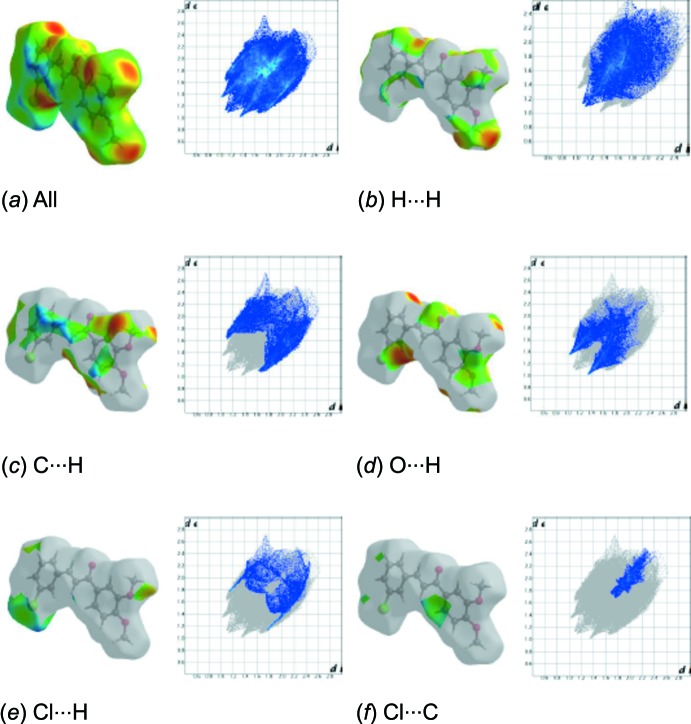
The two-dimensional fingerprint plots of the title compound, showing (*a*) all inter­actions, and delineated into (*b*) H⋯H, (*c*) C⋯H, (*d*) O⋯H, (*e*) Cl⋯H and (*f*) Cl⋯C inter­actions [*d*_e_ and *d*_i_ represent the distances from a point on the Hirshfeld surface to the nearest atoms outside (external) and inside (inter­nal) the surface, respectively].

The overall two-dimensional fingerprint plot for the title compound and those delineated into H⋯H, C⋯H/H⋯C, H⋯O/O⋯H, Cl⋯H/H⋯Cl and Cl⋯C/C⋯Cl contacts are illustrated in Fig. 5 ▸; the percentage contributions from the different inter­atomic contacts to the Hirshfeld surfaces are as follows: H⋯H (36.2%), C⋯H/H⋯C (24.6%), H⋯O/O⋯H (19.2%), Cl⋯H/H⋯Cl (10.5%), Cl⋯C/C⋯Cl (5.8%), C⋯C (3.3%), Cl⋯O/O⋯Cl (0.3%) and O⋯C/C⋯O (0.2%), as shown in the two-dimensional fingerprint plots in Fig. 4 ▸.

## Synthesis and crystallization   

The reagents and solvents for the synthesis were obtained from the Aldrich Chemical Co. and were used without additional purification. 1-(3,4-Di­meth­oxy­phen­yl) ethanone (0.01 mol) and 3-chloro­benzaldehyde (0.01 mol) were dissolved in 20 ml methanol. A catalytic amount of NaOH was added to the solution dropwise with vigorous stirring. The reaction mixture was stirred for about 5–6 h at room temperature. The progress of the reaction was monitored by TLC. The formed crude products were filtered, washed successively with distilled water and recrystallized from ethanol to get the title chalcone. Crystals suitable for X-ray diffraction studies were obtained from acetone solution by slow evaporation at room temperature. The melting point (371–373 K) was determined by a Stuart Scientific (UK) apparatus. The purity of the compound was confirmed by thin layer chromatography using Merck silica gel 60 F254 coated aluminum plates.

## Refinement   

Crystal data, data collection and structure refinement details are summarized in Table 3 ▸. C-bound H atoms were positioned geometrically and refined using a riding model, with C—H = 0.93 Å and *U*_iso_(H) = 1.2*U*_eq_(C) for C—H and C—H = 0.96 Å and U_iso_(H) = 1.5*U*_eq_(C) for methyl H atoms.

## Supplementary Material

Crystal structure: contains datablock(s) global, I. DOI: 10.1107/S205698901800837X/xu5927sup1.cif

Structure factors: contains datablock(s) I. DOI: 10.1107/S205698901800837X/xu5927Isup2.hkl

Supporting information file. DOI: 10.1107/S205698901800837X/xu5927Isup3.cml

CCDC reference: 1847705

Additional supporting information:  crystallographic information; 3D view; checkCIF report

## Figures and Tables

**Table 1:** Hydrogen-bond geometry (Å, °)

*D*—H⋯*A*	*D*—H	H⋯*A*	*D*⋯*A*	*D*—H⋯*A*
C11—H11*A*⋯O3^i^	0.93	2.54	3.417 (2)	157
C15—H15*A*⋯O2^ii^	0.93	2.54	3.4378 (18)	163

Symmetry codes: (i) 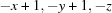; (ii) 
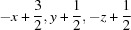
.

**Table 2:** Summary of short inter­atomic contacts (Å) in the title compound

Contact	Distance	Symmetry operation
Cl1⋯H17*B*	3.05	−1 + *x*, 1 + *y*, *z*
Cl1⋯C1	3.4666 (15)	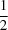 − *x*, 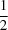 + *y*, 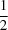 − *z*
O2⋯H15*A*	2.54	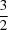 − *x*, − 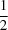 + *y*, 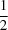 − *z*
O1⋯H17*A*	2.86	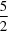 − *x*, 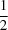 + *y*, 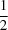 − *z*
H17*C*⋯C10	2.88	1 + *x*, *y*, *z*
H11*A*⋯O3	2.54	1 − *x*, 1 − *y*, −*z*
C1⋯Cl1	3.4666 (15)	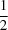 − *x*, − 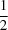 + *y*, 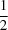 − *z*
H15*A*⋯O2	2.54	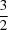 − *x*, 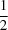 + *y*, 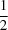 − *z*
C10⋯H17*C*	2.88	−1 + *x*, *y*, *z*
C13⋯C13	3.497 (2)	−*x*, 2 − *y*, −*z*
H13*A*⋯H16*A*	2.46	− 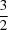 + *x*, 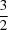 − *y*, − 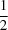 + *z*
H16*A*⋯H13*A*	2.46	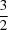 + *x*, 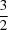 − *y*, 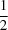 + *z*
H17*A*⋯O1	2.86	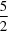 − *x*, − 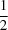 + *y*, 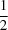 − *z*
H17*B*⋯Cl1	3.05	1 + *x*, −1 + *y*, *z*

**Table 3:** Experimental details

Crystal data	
Chemical formula	C_17_H_15_ClO_3_
*M*_r_	302.74
Crystal system, space group	Monoclinic, *P*2_1_/*n*
Temperature (K)	294
*a*, *b*, *c* (Å)	9.0491 (4), 8.3257 (4), 20.2857 (9)
β (°)	99.484 (1)
*V* (Å^3^)	1507.44 (12)
*Z*	4
Radiation type	Mo *K*α
μ (mm^−1^)	0.26
Crystal size (mm)	0.40 × 0.24 × 0.19

Data collection	
Diffractometer	Bruker APEXII CCD
No. of measured, independent and observed [*I* > 2σ(*I*)] reflections	39332, 5506, 3732
*R*_int_	0.036
(sin θ/λ)_max_ (Å^−1^)	0.758

Refinement	
*R*[*F* ^2^ > 2σ(*F* ^2^)], *wR*(*F* ^2^), *S*	0.049, 0.158, 1.01
No. of reflections	5506
No. of parameters	190
H-atom treatment	H-atom parameters constrained
Δρ_max_, Δρ_min_ (e Å^−3^)	0.31, −0.43

Computer programs: *APEX2* and *SAINT* (Bruker, 2007 ▸), *SHELXS97* (Sheldrick, 2008 ▸), *SHELXL2014* (Sheldrick, 2015 ▸), *ORTEP-3 for Windows* (Farrugia, 2012 ▸) and *PLATON* (Spek, 2009 ▸).

## supplementary crystallographic information

### Crystal data

**Table:**

C_17_H_15_ClO_3_	*F*(000) = 632
*M**_r_* = 302.74	*D*_x_ = 1.334 Mg m^−^^3^
Monoclinic, *P*2_1_/*n*	Mo *K*α radiation, λ = 0.71073 Å
Hall symbol: -P 2yn	Cell parameters from 8862 reflections
*a* = 9.0491 (4) Å	θ = 2.7–30.8°
*b* = 8.3257 (4) Å	µ = 0.26 mm^−^^1^
*c* = 20.2857 (9) Å	*T* = 294 K
β = 99.484 (1)°	Block, yellow
*V* = 1507.44 (12) Å^3^	0.40 × 0.24 × 0.19 mm
*Z* = 4	

### Data collection

**Table:**

Bruker APEXII CCD diffractometer	*R*_int_ = 0.036
φ and ω scans	θ_max_ = 32.6°, θ_min_ = 2.0°
39332 measured reflections	*h* = −13→13
5506 independent reflections	*k* = −12→12
3732 reflections with *I* > 2σ(*I*)	*l* = −30→30

### Refinement

**Table:**

Refinement on *F*^2^	0 restraints
Least-squares matrix: full	Hydrogen site location: inferred from neighbouring sites
*R*[*F*^2^ > 2σ(*F*^2^)] = 0.049	H-atom parameters constrained
*wR*(*F*^2^) = 0.158	*w* = 1/[σ^2^(*F*_o_^2^) + (0.0758*P*)^2^ + 0.3034*P*] where *P* = (*F*_o_^2^ + 2*F*_c_^2^)/3
*S* = 1.01	(Δ/σ)_max_ = 0.001
5506 reflections	Δρ_max_ = 0.31 e Å^−^^3^
190 parameters	Δρ_min_ = −0.43 e Å^−^^3^

### Special details

**Table:**

Geometry. Bond distances, angles etc. have been calculated using the rounded fractional coordinates. All su's are estimated from the variances of the (full) variance-covariance matrix. The cell esds are taken into account in the estimation of distances, angles and torsion angles
Refinement. Refinement on F^2^ for ALL reflections except those flagged by the user for potential systematic errors. Weighted R-factors wR and all goodnesses of fit S are based on F^2^, conventional R-factors R are based on F, with F set to zero for negative F^2^. The observed criterion of F^2^ > 2sigma(F^2^) is used only for calculating -R-factor-obs etc. and is not relevant to the choice of reflections for refinement. R-factors based on F^2^ are statistically about twice as large as those based on F, and R-factors based on ALL data will be even larger.

### Fractional atomic coordinates and isotropic or equivalent isotropic displacement parameters (Å^2^)

**Table:**

	*x*	*y*	*z*	*U*_iso_*/*U*_eq_	
Cl1	−0.02901 (5)	1.02517 (6)	0.15452 (2)	0.0710 (2)	
O1	1.07019 (12)	0.65283 (15)	0.35402 (5)	0.0576 (3)	
O2	1.13649 (12)	0.52216 (16)	0.24887 (6)	0.0634 (4)	
O3	0.67255 (12)	0.57110 (17)	0.07167 (5)	0.0632 (4)	
C1	0.72153 (15)	0.73479 (17)	0.23869 (7)	0.0458 (4)	
C2	0.82535 (15)	0.73642 (17)	0.29717 (7)	0.0463 (4)	
C3	0.96267 (14)	0.66310 (15)	0.29901 (6)	0.0420 (3)	
C4	0.99836 (14)	0.59071 (16)	0.24086 (6)	0.0415 (3)	
C5	0.89499 (14)	0.59023 (15)	0.18335 (6)	0.0405 (3)	
C6	0.75372 (13)	0.66142 (14)	0.18154 (6)	0.0394 (3)	
C7	0.64331 (14)	0.64884 (17)	0.11929 (7)	0.0444 (4)	
C8	0.49469 (15)	0.72588 (18)	0.11575 (7)	0.0495 (4)	
C9	0.38379 (15)	0.69300 (18)	0.06640 (6)	0.0452 (4)	
C10	0.22936 (14)	0.75164 (16)	0.05882 (6)	0.0424 (3)	
C11	0.12555 (17)	0.69543 (19)	0.00574 (7)	0.0525 (4)	
C12	−0.02283 (18)	0.7421 (2)	−0.00148 (8)	0.0610 (5)	
C13	−0.07040 (16)	0.8446 (2)	0.04383 (8)	0.0564 (5)	
C14	0.03261 (16)	0.90063 (17)	0.09633 (7)	0.0470 (4)	
C15	0.18120 (15)	0.85720 (16)	0.10468 (7)	0.0447 (3)	
C16	1.0326 (2)	0.7046 (3)	0.41599 (8)	0.0826 (7)	
C17	1.1814 (2)	0.4431 (2)	0.19409 (9)	0.0669 (6)	
H1A	0.62888	0.78375	0.23788	0.0550*	
H2A	0.80242	0.78689	0.33512	0.0560*	
H5A	0.91867	0.54216	0.14504	0.0490*	
H8A	0.47900	0.79854	0.14872	0.0590*	
H9A	0.40642	0.62539	0.03295	0.0540*	
H11A	0.15612	0.62577	−0.02523	0.0630*	
H12A	−0.09113	0.70376	−0.03734	0.0730*	
H13A	−0.17036	0.87545	0.03911	0.0680*	
H15A	0.24886	0.89746	0.14035	0.0540*	
H16A	1.11764	0.69130	0.45069	0.1240*	
H16B	1.00432	0.81587	0.41282	0.1240*	
H16C	0.95044	0.64175	0.42628	0.1240*	
H17A	1.28064	0.40096	0.20705	0.1000*	
H17B	1.11330	0.35669	0.17988	0.1000*	
H17C	1.18073	0.51778	0.15799	0.1000*	

### Atomic displacement parameters (Å^2^)

**Table:**

	*U*^11^	*U*^22^	*U*^33^	*U*^12^	*U*^13^	*U*^23^
Cl1	0.0650 (3)	0.0704 (3)	0.0809 (3)	0.0083 (2)	0.0216 (2)	−0.0176 (2)
O1	0.0518 (6)	0.0735 (7)	0.0440 (5)	0.0124 (5)	−0.0028 (4)	−0.0031 (5)
O2	0.0487 (6)	0.0881 (8)	0.0518 (6)	0.0278 (5)	0.0039 (4)	−0.0044 (5)
O3	0.0479 (6)	0.0898 (8)	0.0505 (6)	0.0088 (5)	0.0038 (4)	−0.0226 (6)
C1	0.0400 (6)	0.0500 (7)	0.0472 (7)	0.0080 (5)	0.0064 (5)	−0.0052 (5)
C2	0.0462 (7)	0.0506 (7)	0.0420 (6)	0.0063 (5)	0.0074 (5)	−0.0069 (5)
C3	0.0425 (6)	0.0424 (6)	0.0398 (6)	0.0012 (5)	0.0028 (5)	0.0021 (5)
C4	0.0382 (6)	0.0423 (6)	0.0441 (6)	0.0057 (5)	0.0073 (5)	0.0033 (5)
C5	0.0404 (6)	0.0425 (6)	0.0396 (6)	0.0032 (5)	0.0093 (5)	−0.0010 (5)
C6	0.0382 (6)	0.0393 (5)	0.0403 (6)	0.0007 (4)	0.0057 (4)	−0.0005 (4)
C7	0.0384 (6)	0.0510 (7)	0.0434 (6)	0.0014 (5)	0.0055 (5)	−0.0046 (5)
C8	0.0426 (6)	0.0562 (8)	0.0476 (7)	0.0070 (6)	0.0014 (5)	−0.0090 (6)
C9	0.0424 (6)	0.0539 (7)	0.0388 (6)	0.0028 (5)	0.0050 (5)	−0.0017 (5)
C10	0.0401 (6)	0.0469 (6)	0.0384 (6)	−0.0001 (5)	0.0011 (5)	0.0006 (5)
C11	0.0493 (7)	0.0603 (8)	0.0449 (7)	0.0006 (6)	−0.0014 (6)	−0.0093 (6)
C12	0.0468 (8)	0.0733 (10)	0.0568 (8)	−0.0030 (7)	−0.0092 (6)	−0.0092 (7)
C13	0.0379 (6)	0.0642 (9)	0.0643 (9)	0.0002 (6)	−0.0002 (6)	0.0022 (7)
C14	0.0452 (7)	0.0445 (6)	0.0519 (7)	−0.0005 (5)	0.0094 (5)	0.0004 (5)
C15	0.0419 (6)	0.0476 (6)	0.0427 (6)	−0.0022 (5)	0.0016 (5)	−0.0030 (5)
C16	0.0814 (12)	0.1149 (17)	0.0452 (8)	0.0267 (12)	−0.0080 (8)	−0.0161 (10)
C17	0.0583 (9)	0.0781 (11)	0.0673 (10)	0.0238 (8)	0.0189 (8)	−0.0001 (8)

### Geometric parameters (Å, º)

**Table:**

Cl1—C14	1.7310 (15)	C12—C13	1.374 (2)
O1—C3	1.3570 (16)	C13—C14	1.376 (2)
O1—C16	1.422 (2)	C14—C15	1.376 (2)
O2—C4	1.3593 (17)	C1—H1A	0.9300
O2—C17	1.408 (2)	C2—H2A	0.9300
O3—C7	1.2273 (18)	C5—H5A	0.9300
C1—C2	1.387 (2)	C8—H8A	0.9300
C1—C6	1.3832 (18)	C9—H9A	0.9300
C2—C3	1.3794 (19)	C11—H11A	0.9300
C3—C4	1.4088 (17)	C12—H12A	0.9300
C4—C5	1.3698 (17)	C13—H13A	0.9300
C5—C6	1.4041 (17)	C15—H15A	0.9300
C6—C7	1.4792 (18)	C16—H16A	0.9600
C7—C8	1.4810 (19)	C16—H16B	0.9600
C8—C9	1.3241 (19)	C16—H16C	0.9600
C9—C10	1.4643 (19)	C17—H17A	0.9600
C10—C11	1.3884 (19)	C17—H17B	0.9600
C10—C15	1.4005 (19)	C17—H17C	0.9600
C11—C12	1.382 (2)		
			
C3—O1—C16	117.66 (12)	C6—C1—H1A	120.00
C4—O2—C17	118.77 (12)	C1—C2—H2A	120.00
C2—C1—C6	120.98 (13)	C3—C2—H2A	120.00
C1—C2—C3	119.94 (13)	C4—C5—H5A	120.00
O1—C3—C2	124.80 (12)	C6—C5—H5A	120.00
O1—C3—C4	115.43 (11)	C7—C8—H8A	119.00
C2—C3—C4	119.77 (12)	C9—C8—H8A	119.00
O2—C4—C3	114.38 (11)	C8—C9—H9A	116.00
O2—C4—C5	125.88 (12)	C10—C9—H9A	117.00
C3—C4—C5	119.72 (12)	C10—C11—H11A	120.00
C4—C5—C6	120.80 (11)	C12—C11—H11A	120.00
C1—C6—C5	118.76 (11)	C11—C12—H12A	120.00
C1—C6—C7	122.74 (11)	C13—C12—H12A	120.00
C5—C6—C7	118.45 (11)	C12—C13—H13A	121.00
O3—C7—C6	120.42 (12)	C14—C13—H13A	121.00
O3—C7—C8	120.14 (13)	C10—C15—H15A	120.00
C6—C7—C8	119.40 (12)	C14—C15—H15A	120.00
C7—C8—C9	121.08 (13)	O1—C16—H16A	109.00
C8—C9—C10	127.00 (13)	O1—C16—H16B	109.00
C9—C10—C11	118.76 (12)	O1—C16—H16C	109.00
C9—C10—C15	122.38 (12)	H16A—C16—H16B	109.00
C11—C10—C15	118.81 (12)	H16A—C16—H16C	109.00
C10—C11—C12	120.58 (14)	H16B—C16—H16C	109.00
C11—C12—C13	120.63 (15)	O2—C17—H17A	109.00
C12—C13—C14	118.77 (14)	O2—C17—H17B	109.00
Cl1—C14—C13	118.53 (11)	O2—C17—H17C	109.00
Cl1—C14—C15	119.45 (11)	H17A—C17—H17B	109.00
C13—C14—C15	122.00 (13)	H17A—C17—H17C	110.00
C10—C15—C14	119.21 (13)	H17B—C17—H17C	109.00
C2—C1—H1A	120.00		
			
C16—O1—C3—C2	7.7 (2)	C1—C6—C7—C8	3.84 (19)
C16—O1—C3—C4	−171.77 (15)	C5—C6—C7—O3	3.5 (2)
C17—O2—C4—C3	178.44 (13)	C5—C6—C7—C8	−178.75 (12)
C17—O2—C4—C5	0.3 (2)	O3—C7—C8—C9	10.5 (2)
C6—C1—C2—C3	−0.5 (2)	C6—C7—C8—C9	−167.28 (13)
C2—C1—C6—C5	−1.0 (2)	C7—C8—C9—C10	175.76 (13)
C2—C1—C6—C7	176.44 (13)	C8—C9—C10—C11	−175.75 (15)
C1—C2—C3—O1	−177.65 (13)	C8—C9—C10—C15	1.6 (2)
C1—C2—C3—C4	1.8 (2)	C9—C10—C11—C12	177.04 (14)
O1—C3—C4—O2	−0.40 (17)	C15—C10—C11—C12	−0.4 (2)
O1—C3—C4—C5	177.86 (12)	C9—C10—C15—C14	−176.57 (13)
C2—C3—C4—O2	−179.87 (12)	C11—C10—C15—C14	0.8 (2)
C2—C3—C4—C5	−1.60 (19)	C10—C11—C12—C13	−0.2 (2)
O2—C4—C5—C6	178.20 (13)	C11—C12—C13—C14	0.4 (2)
C3—C4—C5—C6	0.2 (2)	C12—C13—C14—Cl1	−178.21 (12)
C4—C5—C6—C1	1.13 (19)	C12—C13—C14—C15	0.0 (2)
C4—C5—C6—C7	−176.39 (12)	Cl1—C14—C15—C10	177.64 (10)
C1—C6—C7—O3	−173.88 (14)	C13—C14—C15—C10	−0.6 (2)

### Hydrogen-bond geometry (Å, º)

**Table:**

*D*—H···*A*	*D*—H	H···*A*	*D*···*A*	*D*—H···*A*
C9—H9*A*···O3	0.93	2.45	2.7888 (18)	102
C11—H11*A*···O3^i^	0.93	2.54	3.417 (2)	157
C15—H15*A*···O2^ii^	0.93	2.54	3.4378 (18)	163

Symmetry codes: (i) −*x*+1, −*y*+1, −*z*; (ii) −*x*+3/2, *y*+1/2, −*z*+1/2.
